# Supplementary material for: Identification and Molecular Characterization of Giant Liver Fluke (Fascioloides magna) Infection in European Fallow Deer (Dama dama) in Romania—First Report
Source: Microorganisms. 2024 Mar 6;12(3):527. doi: 10.3390/microorganisms12030527 (PMC10974814; doi:10.3390/microorganisms12030527)
Supplement: Supplementary file 1 [file microorganisms-12-00527-s001.zip › microorganisms-2894321-supplementary.pdf]

Supplementary Figure S1

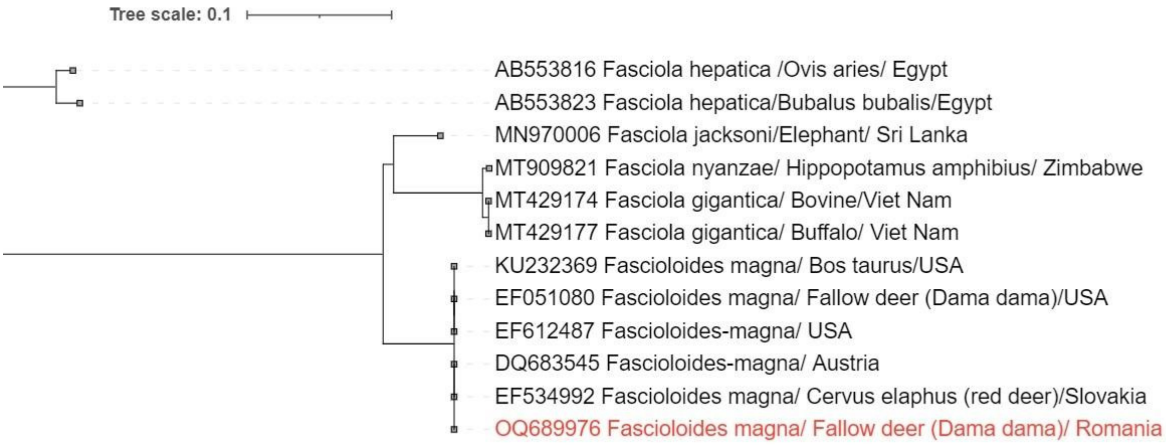

Supplementary Figure S2

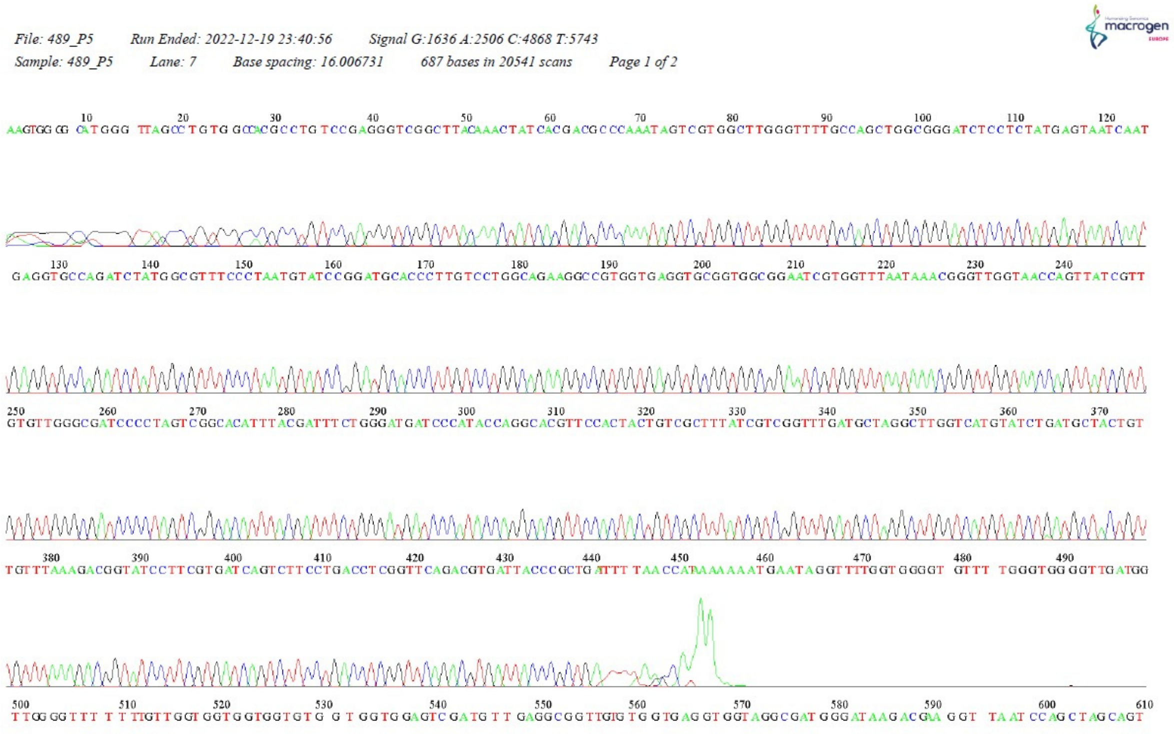

Supplementary Figure S3

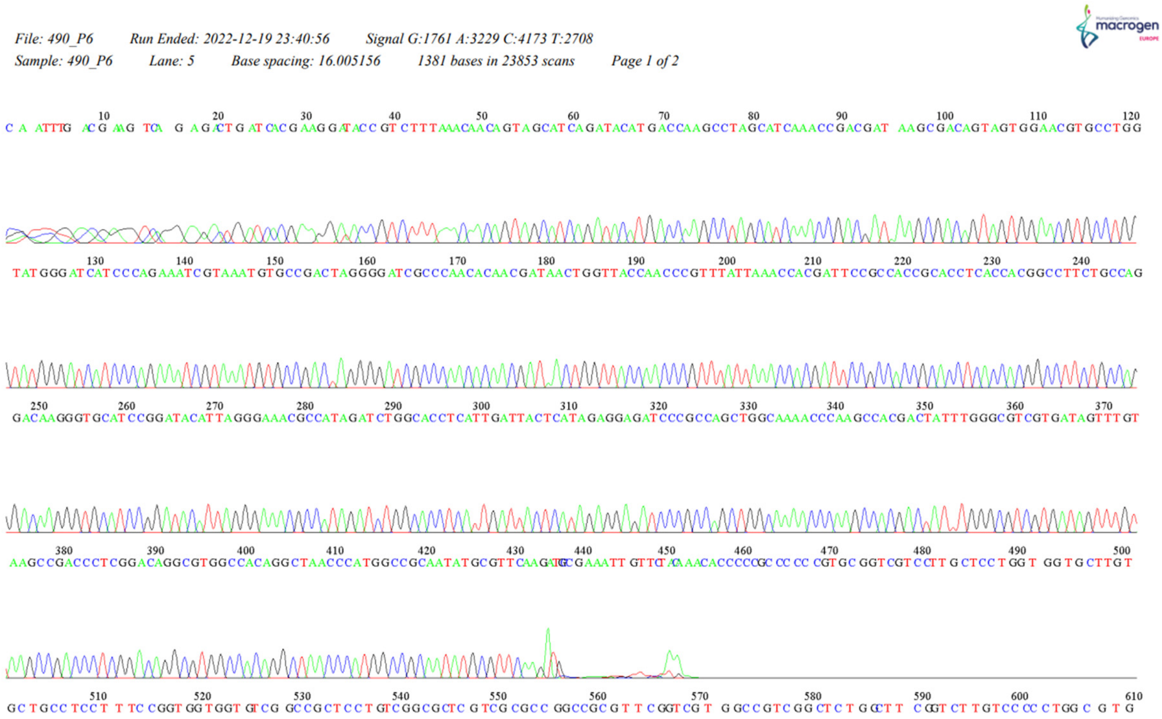

Supplementary Figure S4

An official website of the United States government
Here's how you know

Nucleotide

GenBank

Fascioloides magna isolate RO_489_FM 5.8S ribosomal RNA gene, partial sequence; internal transcribed spacer 2, complete sequence; and large subunit ribosomal RNA gene, partial sequence

GenBank: OQ689976.1

FASTA Graphics

Go to:

```
LOCUS       OQ689976                 428 bp    DNA     linear   INV 29-MAR-2023
DEFINITION  Fascioloides magna isolate RO_489_FM 5.8S ribosomal RNA gene,
            partial sequence; internal transcribed spacer 2, complete sequence;
            and large subunit ribosomal RNA gene, partial sequence.
ACCESSION   OQ689976
VERSION     OQ689976.1
KEYWORDS    .
SOURCE      Fascioloides magna
  ORGANISM  Fascioloides magna
            Eukaryota; Metazoa; Spiralia; Lophotrochozoa; Platyhelminthes;
            Trematoda; Digenea; Plagiorchiida; Echinostomata;
            Echinostomatoidea; Fasciolidae; Fascioloides.
REFERENCE   1  (bases 1 to 428)
  AUTHORS   Imre,M. and Mederle,N.
  TITLE     First report of Fascioloides magna in fallow deer in Romania
  JOURNAL   Unpublished
REFERENCE   2  (bases 1 to 428)
  AUTHORS   Imre,M. and Mederle,N.
  TITLE     Direct Submission
  JOURNAL   Submitted (24-MAR-2023) Parasitology and Parasitic Disease
            Department, University of Life Sciences Timisoara, Timisoara, Calea
            Aradului No. 119, Timisoara, Timis 300645, Romania
COMMENT     ##Assembly-Data-START##
            Sequencing Technology :: Sanger dideoxy sequencing
            ##Assembly-Data-END##
FEATURES             Location/Qualifiers
     source          1..428
                     /organism="Fascioloides magna"
                     /mol_type="genomic DNA"
                     /isolate="RO_489_FM"
                     /isolation_source="liver"
                     /host="Dama dama"
                     /db_xref="taxon:394415"
                     /country="Romania"
                     /collection_date="12-Nov-2022"
     misc_RNA        <1..>428
                     /note="contains 5.8S ribosomal RNA, internal transcribed
                     spacer 2, and large subunit ribosomal RNA"
ORIGIN
        1 tagcctgtgg ccacgcctgt ccgagggtcg gcttacaaac tatcacgacg cccaaatagt
       61 cgtggcttgg gttttgccag ctggcgggat ctcctctatg agtaatcaat gaggtgccag
      121 atctatggcg tttccctaat gtatccggat gcacccttgt cctggcagaa ggccgtggtg
      181 aggtgcggtg gcggaatcgt ggtttaataa acgggttggt aaccagttat cgttgtgttg
      241 ggcgatcccc tagtcggcac atttacgatt tctgggatga tcccatacca ggcacgttcc
      301 actactgtcg ctttatcgtc ggtttgatgc taggcttggt catgtatctg atgctactgt
      361 tgtttaaaga cggtatcctt cgtgatcagt cttcctgacc tcggttcaga cgtgattacc
      421 cgctgatt
//
```
